# Patient‐ and proxy‐reported quality of life in advanced dementia with Lewy bodies

**DOI:** 10.1002/alz.13745

**Published:** 2024-02-23

**Authors:** Melissa J. Armstrong, Brian LaBarre, Kaitlin Sovich, Susan M. Maixner, Henry L. Paulson, Carol Manning, Julie A. Fields, Angela Lunde, Leah Forsberg, Bradley F. Boeve, James E. Galvin, Angela S. Taylor, Zhigang Li

**Affiliations:** ^1^ Department of Neurology University of Florida College of Medicine Gainesville Florida USA; ^2^ Norman Fixel Institute for Neurological Diseases Gainesville Florida USA; ^3^ Department of Biostatistics University of Florida College of Medicine Gainesville Florida USA; ^4^ Department of Psychiatry University of Michigan Ann Arbor Michigan USA; ^5^ Department of Neurology University of Michigan Ann Arbor Michigan USA; ^6^ Department of Neurology University of Virginia Charlottesville Virginia USA; ^7^ Department of Psychiatry and Psychology Mayo Clinic Rochester Minnesota USA; ^8^ Department of Neurology Mayo Clinic Rochester Minnesota USA; ^9^ Comprehensive Center for Brain Health Department of Neurology University of Miami Miller School of Medicine Miami Florida USA; ^10^ Lewy Body Dementia Association Lilburn Georgia USA

**Keywords:** caregivers, dementia, dementia with Lewy bodies, Lewy body disease, quality of life

## Abstract

**INTRODUCTION:**

Little is known regarding quality of life (QoL) in dementia with Lewy bodies (DLB), particularly in advanced stages.

**METHODS:**

Dyads of individuals with moderate–advanced DLB and their primary caregivers were recruited from specialty clinics, advocacy organizations, and research registries. The study collected demographics, disease‐related measures, and measures of patient/caregiver experiences.

**RESULTS:**

The Quality of Life in Alzheimer's Disease (QoL‐AD) was completed by the person with DLB and the caregiver (proxy) in 61 dyads; 85 dyads had only a proxy‐completed QoL‐AD. Patient‐ and proxy‐reported scores were moderately correlated (*r* = 0.57, *P* < 0.0001). Worse patient‐reported QoL correlated with daytime sleepiness, autonomic symptom burden, and behavioral symptoms. Proxy ratings correlated with dementia severity, daytime sleepiness, behavioral symptoms, dependence in activities of daily living, and caregiver experience measures.

**DISCUSSION:**

Patient‐ and proxy‐reported quality of life (QoL) should be assessed separately in advanced DLB. Some symptoms associated with QoL have available therapeutic options. Research is needed regarding strategies to optimally improve QoL in DLB.

**Highlights:**

- Patient and proxy quality of life (QoL) ratings had moderate correlation in advanced dementia with Lewy bodies.
- Daytime sleepiness affected patient‐ and proxy‐reported QoL.
- Behavioral symptoms affected patient‐ and proxy‐reported QoL.
- Autonomic symptom burden affected patient‐reported QoL.
- Dementia severity, dependence, and caregiver experiences affected proxy ratings.

## 1 BACKGROUND

Dementia with Lewy bodies (DLB) is one of the most common degenerative dementias after Alzheimer's disease (AD) dementia. Despite DLB prevalence, evidence regarding the quality of life (QoL) of individuals living with DLB is limited. Prior studies suggest that patient QoL is worse in DLB compared to AD dementia,^1^, ^2^, ^3^, ^4^ but limitations include small sample sizes, inclusion of only individuals with early DLB or a wide range of DLB severities, reliance on proxy reporting, use of various instruments, and publication only in abstract form.

Three prior studies specifically queried patient‐reported QoL in individuals with DLB, one of which was reported in two publications.^1^, ^4^, ^5^, ^6^ In a study of correlates of patient‐reported QoL in early DLB (mean Mini‐Mental State Examination [MMSE] score 25, range 22–27), worse QoL correlated with more depressive symptoms and higher dependency in instrumental activities of daily living (IADLs).^4^ In the DEvELOP cohort including individuals with both mild cognitive impairment and dementia (mean MMSE 25 ± 3), analyses of QoL used a weighted Quality of Life in Alzheimer's Disease (QoL‐AD) composite score calculated by combining patient‐ and caregiver‐rated QoL‐AD scores, weighting the patient‐rated score twice as much.^5^ Using this composite score, hallucinations, constipation, depressive symptoms, and higher IADL dependency were related to lower QoL in univariate analyses, but hallucinations were no longer significant in the multivariable model.^5^ In a subsequent longitudinal analysis with a larger cohort, declining QoL (rater not specified) correlated with changes in depressive symptoms, but this was not significant after adjustment for multiple comparisons.^6^ In the other study including both patient‐ and proxy‐rated QoL, regression analyses were only performed using proxy‐rated scores.^1^ Features associated with proxy‐reported QoL for individuals with DLB included neuropsychiatric symptoms, IADL dependence, and whether the person with DLB lived with the primary caregiver.^1^ In a database study available only in abstract form (in which the individual completing the forms was not documented), QoL in DLB correlated most strongly with neuropsychiatric symptoms, cognition, and one's ability to perform activities of daily living (ADLs).^7^

Research aiming to improve QoL is a priority of individuals living with DLB and their caregivers.^8^ Particularly given the limitations in existing literature, further investigation into drivers of QoL is critical, particularly to inform potential interventions for improving QoL. The current analysis investigates correlates of patient‐ and caregiver/proxy‐reported QoL in moderate–advanced DLB stages using results from an ongoing multisite observational cohort study.

## 2 METHODS

### 2.1 Study design and recruitment

The current analysis uses baseline visit data from the ongoing PACE‐DLB (Identifying Factors Predicting ACcurately End‐of‐Life in Dementia with Lewy Bodies and Promoting Quality End‐of‐Life Experiences) study.^9^ PACE‐DLB is an observational longitudinal cohort study involving five Lewy Body Dementia Association (LBDA) Research Centers of Excellence in the United States (University of Florida, University of Michigan, Mayo Clinic Rochester, University of Virginia, University of Miami) and recruitment of a virtual cohort to include participants not receiving subspecialty care. Recruitment for the virtual cohort occurred through the LBDA, Lewy Body Dementia Research Center, ClinicalTrials.gov, Alzheimer's Association Trial Match, the Fox Trial Finder, advertising in caregiver newsletters, support group presentations, and unaffiliated neurologists who learned about the study and offered to share study recruitment materials.

### 2.2 Study participants

Inclusion criteria were: (1) patient and primary informal caregiver willing to participate as a dyad, (2) US residents, (3) patient with a clinical diagnosis of DLB, (4) patient with at least moderate severity dementia as assessed by the Quick Dementia Rating System (QDRS, score of > 12 suggestive of moderate dementia or ≥ 3 domains rated at least moderate severity^10^), (5) patient expected to live at least 6 months, and (6) caregiver Telephone Interview for Cognitive Status score of > 31 to ensure that the caregiver was able to reliably complete study visits.^11^ Patient‐ or clinician‐reported diagnosis of DLB was supported using the patient and family version of the Lewy Body Composite Risk Score (LBCRS). The LBCRS is a 10‐item questionnaire that covers core and supportive features of DLB that is able to discriminate cognitive impairment due to DLB from other causes of cognitive impairment.^12^ An LBCRS score ≥3 is supportive of DLB and was required for enrollment. The participating caregiver was the person providing the majority of the patient's informal care (whether the individual with DLB was living at home or in a facility) and attending the majority of the patient's clinical visits. Caregivers provided written informed consent for their own participation and patients provided consent or assent with additional consent from a legally authorized representative. The study was approved by the University of Florida institutional review board (IRB202001438) and registered on ClinicalTrials.gov (NCT04829656).

### 2.3 Study measures

Quality of life of the individual with DLB was assessed using patient‐ and proxy‐completed versions of the 13‐item QoL‐AD scale, validated for individuals with dementia.^13^ QoL‐AD scores range from 13 to 52 with higher scores indicating better QoL.^13^ All study measures were caregiver‐completed except the patient‐reported QoL‐AD. Demographics included patient and caregiver gender, sex, age, racial/ethnic background, education, disease duration, hours spent caregiving, and the relationship of the caregiver to the individual with DLB. Sex and gender were congruent for most participants; gender was used as the variable for the QoL analyses.

**RESEARCH IN CONTEXT**

1. **Systematic review**: The authors reviewed the literature regarding quality of life (QoL) in dementia with Lewy bodies (DLB) using traditional (e.g., PubMed) sources. Worse QoL correlated with psychiatric symptoms and dependency in instrumental activities of daily living in multiple studies, but most research relied on proxy reporting and investigated early disease stages.
2. **Interpretation**: This is the first study investigating QoL in moderate–advanced DLB. Patient‐ and proxy‐reported scores were moderately correlated, suggesting the need to assess these separately. Worse patient‐reported QoL correlated with daytime sleepiness, autonomic symptom burden, and behavioral symptoms. Proxy ratings correlated with dementia severity, daytime sleepiness, behavioral symptoms, dependence in activities of daily living, and caregiver experience measures.
3. **Future directions**: Future research should investigate QoL along the continuum of DLB and identify pharmacologic and non‐pharmacologic strategies to target features associated with lower QoL.

For all scales, unless otherwise noted, higher scores represent more impairment and/or symptom burden. The LBCRS measured the breadth of DLB symptoms present (scale score 0–10, but ≥ 3 required for enrollment).^12^ While designed as a screening instrument rather than a measure of disease severity, this interview‐based scale provided a way to assess disease scope using an instrument that could be completed verbally. Cognitive function was assessed via the QDRS (score 0–30) from which a Clinical Dementia Rating (CDR) Dementia Staging Instrument score and its sum of boxes score (CDR‐SB) can be reliably estimated.^10^ The Neuropsychiatric Inventory Questionnaire (NPI‐Q) assessed behavioral symptoms (severity score 0–36).^14^, ^15^ The Mayo Fluctuations Scale (score 0–4)^16^ and Clinician Assessment of Fluctuations (severity score 0–16)^17^ measured fluctuations. The Mayo Sleep Questionnaire (REM sleep behavior disorder dichotomized as yes/no based on question 1; alertness rating 0–10 with lower scores indicating more sleepiness and higher scores indicating more wakefulness)^18^ and Epworth Sleepiness Scale (score 0–24)^19^ assessed aspects of sleep relevant to DLB. The Autonomic Systems Checklist (score 0–19) from the National Alzheimer's Coordinating Center Lewy body dementia module identified the presence of autonomic symptoms.^20^ Activities of daily living were assessed as part of the Advanced Dementia Prognostic Tool (ADEPT) and reflected seven ADLs each scored on a 0 to 4 point scale (total 0–28).^21^, ^22^ The Charlson Comorbidity Index (CCI) provided a measure of comorbidities^23^ and the Anticholinergic Cognitive Burden (ACB) scale assessed the potential impact of anticholinergic medications on cognition.^24^ ^.^ ^25^

Caregiver depression was measured with the Center for Epidemiologic Studies Depression Scale (scores 0–60).^26^ The shortened Zarit Burden Interview (sZBI) assessed caregiver burden (scores 0–48).^27^ The Meuser–Marwit Caregiver Grief Inventory—Short Form, designed for caregivers of individuals with dementia, assessed anticipatory grief (scores 18–90).^28^ The Multidimensional Scale of Perceived Social Support (MSPSS) assessed family, friend, and significant other support (12 items rated on a 1–7 Likert scale including three levels of disagreement, one neutral option, and three levels of agreement; subscale and total scores represent a mean of contributing items).^29^, ^30^ The Resilience Scale assessed caregiver resilience.^31^ The Revised Scale for Caregiving Self‐Efficacy covered topics relating to confidence obtaining respite, responding to disruptive behaviors, and controlling upsetting thoughts about caregiving. Each subscale assessed five items ranked by caregivers on a 0–100 point scale (higher score indicates that the caregiver is more certain they can do the behavior). Subscale scores reflected the mean of the five related questions.^32^, ^33^ Coping strategies were measured by the Brief Coping Orientation to Problems Experienced, which assessed 28 coping behaviors each rated from 1 (I haven't been doing this at all) to 4 (I've been doing this a lot).^34^ Items were separated into three subscales used in prior dementia caregiving research: problem‐focused strategies (active coping, instrumental support, planning; score 6–24), emotion‐focused strategies (acceptance, emotional support, humor, positive reframing, religion; score 10–40), and dysfunctional coping strategies (behavioral disengagement, denial, self‐distraction, self‐blame, substance use, venting; score 12–48).^35^ Caregivers also completed the QoL‐AD on their own behalf (i.e., as a measure of their own QoL).

### 2.4 Study visits

Study visits were designed for virtual and in‐person completion given a priori recruitment of a virtual cohort. Because funding occurred during the COVID pandemic, dyads enrolled at LBDA Research Centers of Excellence were allowed to choose in‐person or virtual study completion. Virtual study visits were completed by phone or secure videoconferencing. Measures were administered verbally to minimize differences in conduct between the virtual and in‐person groups. Verbal administration was used across measures because some older caregivers could be uncomfortable with self‐administered electronic form completion. Visits were completed over multiple days if needed. Coordinators entered data directly into electronic case report forms using REDCap (Research Electronic Data Capture) tools hosted at the University of Florida.^36^, ^37^

### 2.5 Analysis

Results for the current analysis reflect data collected through December 8, 2022. Sample size estimates for the PACE‐DLB study were based on the aims for the main study, which is still in progress. Descriptive statistical measures included mean, median, standard deviation (SD), minimum and maximum values of continuous variables, and frequency of categorical variables. Analyses were performed using available data for each question; if data were missing, the total “*n*” for that item or analysis was noted. Concordance between patient‐ and proxy‐reported QoL was assessed using Spearman correlation. The inter‐rater reliability measure Kappa was not used because each dyad was rating QoL for a unique individual with DLB, which was not rated by other raters.^38^ Non‐parametric methods including Spearman correlation, two‐sample Wilcoxon test, and multi‐group Kruskal–Wallis test assessed factors associated with patient QoL. Because the goal of the analysis was to identify factors associated with QoL, particularly those that could be targeted with clinical interventions, the primary analysis focused on univariate correlations. Multivariable post hoc analyses were subsequently performed using linear regression models with backward model selections using Schwarz Bayesian information criterion.

For correlations, the strength of association was assessed as very weak if *r* < 0.20, weak if 0.2 to 0.39, moderate if 0.4 to 0.59, strong if 0.6 to 0.79, and very strong if 0.8 to 1. Multiple testing was adjusted by the false discovery rate (FDR) method^39^ and all presented *P* values are adjusted *P* values. Some adjusted *P* values are the same due to the discrete property of the FDR method. Results were considered statistically significant if the adjusted *P* value was < 0.05. For results using the proxy‐completed QoL‐AD scores, sensitivity analyses were performed looking separately at the cohort with QoL‐AD scores completed by both the person with DLB and the proxy (caregiver) versus the cohort with only proxy‐completed QoL‐AD scores.

## 3 RESULTS

The current analysis included the first 146 dyads with a baseline visit. Of the 146 visits, 78 (53.4%) were completed by phone, 62 (42.5%) by web conferencing, 5 (3.42%) in person, and 1 (0.68%) mixed (in person and remote). Individuals with DLB had a mean age of 74.8 years and the majority were men (78.8%) and identified as White non‐Hispanic. Most participants (89.7%) lived at home at the time of the study visit (Table 1). Caregivers were mostly women, reflecting both wives and daughters, and 87.7% of caregivers lived with the person with DLB (Table 2).

**TABLE 1:** Baseline characteristics of individuals with DLB in the PACE‐DLB cohort.

	Individuals with DLB (*n* = 146)
Age (years; mean, SD)	74.83 (7.9)
Sex (% male)	115 (78.77%)
Disease duration at baseline (years; mean, SD)	3.06 (2.46)
Race	
White	140 (95.89%)
Black or African American	2 (1.37%)
Asian	2 (1.37%)
More than one race	1 (0.68%)
Unknown or not reported	1 (0.68%)
Ethnicity	
Hispanic	3 (2.05%)
Non‐Hispanic	143 (97.95%)
Living situation	
Home	131 (89.73%)
Assisted living	7 (4.79%)
Nursing home	8 (5.48%)
LBCRS score (mean, SD)	8.16 (1.58)
Total QDRS score (mean, SD)	18.25 (4.79)
Dementia severity based on QDRS score	
Moderate (score 13–20.5)	104 (71.2%)
Severe (score 21–30)	42 (28.8%)
CDR sum of boxes (mean, SD)	12.03 (3.17)
Fluctuations (*n*, %)	122 (83.56%)
Fluctuation severity (mean, SD)	8.77 (6.68)
RBD symptoms (*n*, %)	94/128^a^ (73.44%)
Hallucinations (*n*, %)	88 (60.27%)
Excessive daytime sleepiness (*n*, %)	104 (71.23%)
Excessive daytime sleepiness (ESS score; mean, SD)	13.71 (5.47)
Mayo Sleep Questionnaire Alertness score (mean, SD)	5.37 (2.13)^a^
Parkinsonism (at least one reported feature; *n*, %)	143 (97.95%)
Parkinsonism (all four reported features of tremor, slowness, rigidity, postural instability) (*n*, %)	76 (52.05%)
Autonomic symptoms (total score; mean, SD)	8.25 (3.36)
Orthostatic hypotension (*n*, %)	97 (66.44%)
NPI‐Q severity score (mean, SD)	12.86 (5.83)
ADL score (mean, SD)	10.05 (8.07)
Number of medications	8.16 (3.56)
Parkinsonism medications (yes; *n*, %)	70 (47.95%)
Cholinesterase inhibitor (yes; *n*, %)	101 (69.18%)
Antipsychotic (yes; *n*, %)	64 (43.84%)
Antidepressant/anti‐anxiety agent (yes; *n*, %)	88 (60.27%)
Anticholinergic Cognitive Burden scale (mean, SD)	1.82 (1.84)
Charlson Comorbidity Index (mean, SD)	2.05 (1.5)

Abbreviations: ADL, activities of daily living; CDR, Clinical Dementia Rating; DLB, dementia with Lewy bodies; ESS, Epworth Sleepiness Scale; LBCRS, Lewy Body Composite Risk Score; NPI‐Q, Neuropsychiatric Inventory Questionnaire; PACE‐DLB, Identifying Factors Predicting ACcurately End‐of‐Life in Dementia with Lewy Bodies and Promoting Quality End‐of‐Life Experiences study; QDRS, Quick Dementia Rating System score; RBD, REM sleep behavior disorder; SD, standard deviation.

^a^ The total “*n*” for the Mayo Sleep Questionnaire is 128; 18 caregivers reported insufficient knowledge to complete this scale.

**TABLE 2:** Baseline characteristics of caregivers of individuals with DLB in the PACE‐DLB cohort.

	Caregivers (*n* = 146)
Age (years; mean, SD)	67.95 (8.57)
Sex (% male)	19 (13.01%)
Race	
White	138 (94.52%)
Asian	3 (2.05%)
Black or African American	2 (1.37%)
Native Hawaiian or other Pacific Islander	1 (0.68%)
More than one race	1 (0.68%)
Unknown or not reported	1 (0.68%)
Ethnicity	
Hispanic	7 (4.79%)
Non‐Hispanic	139 (95.21%)
Caregiver role	
Wife	104 (71.23%)
Husband	16 (10.96%)
Daughter	19 (13.01%)
Son	1 (0.68%)
Other^a^	6 (4.11%)
Years spent caregiving	4.24 (4.48)
Hours per day “on duty” for caregiving (mean, SD)	17.35 (8.46)
Hours per day must be in same room as loved one (mean, SD)	7.71 (7.58)
Live with patient (yes, %)	128 (87.67%)
Anyone else in home (yes, %)	24 (18.32%)
CES‐D score (mean, SD) (*n* = 145)	14.91 (9.46)
Caregiver burden (sZBI score; mean, SD) (*n* = 145)	18.45 (8.2)
Anticipatory grief (MMCGI total score; mean, SD) (*n* = 145)	56.01 (12.16)
Social support (MSPSS total score; mean, SD) (*n* = 145)	5.41 (1.1)
Caregiver quality of life (QoL‐AD; mean, SD)	39.92 (5.97)
Caregiver resilience (mean, SD) (*n* = 145)	148.87 (14.64)
Self‐efficacy: Controlling Upsetting Thoughts (mean, SD)	73.38 (21.62)
Self‐efficacy: Obtaining Respite (mean, SD) (*n* = 142)	57.84 (30.57)
Self‐efficacy: Responding to Disruptive Behaviors (mean, SD) (*n* = 139)	80.29 (18.43)
Brief COPE: Dysfunctional Coping Strategies (mean, SD) (*n* = 145)	20.56 (4.11)
Brief COPE: Emotion‐Focused Strategies (mean, SD) (*n* = 145)	28.22 (5.64)
Brief COPE: Problem‐Focused Strategies (mean, SD) (*n* = 145)	19.04 (3.83)

Abbreviations: CES‐D, Center for Epidemiologic Studies Depression Scale; COPE, Coping Orientation to Problems Experienced; DLB, dementia with Lewy bodies; MMCGI, Meuser‐Marwit Caregiver Grief Inventory—Short Form; MSPSS, Multidimensional Scale of Perceived Social Support; PACE‐DLB, Identifying Factors Predicting ACcurately End‐of‐Life in Dementia with Lewy Bodies and Promoting Quality End‐of‐Life Experiences study; QoL‐AD, Quality of Life in Alzheimer's Disease scale; SD, standard deviation; sZBI, shortened Zarit Burden interview.

^a^ Other reported roles: 2 siblings, 1 ex‐spouse, 1 significant other.

### 3.1 Patient‐ and proxy‐reported quality of life

The QoL‐AD was completed by both the person with DLB (patient) and the caregiver (proxy) in 61 dyads (41.8%). The caregiver reported that the person with DLB was unable to complete the QoL‐AD in 85 dyads (58.2%), though specific reasons were not systematically captured. Moderate (versus severe) dementia was more common in the 61 dyads with both patient‐ and proxy‐completed scores (52/61, 85.25% vs. 52/85, 61.18%; chi‐square *P* = 0.0015). In the subset with both a patient‐ and proxy‐completed QoL‐AD, the mean patient‐completed QoL‐AD was 34.48 (SD 5.72) versus a mean proxy‐completed QoL‐AD score of 30.87 (SD 4.55). For dyads in which only the caregiver completed the QoL‐AD (*n* = 85), the mean score was 28.47 (SD 5.23). For the 61 dyads with both QoL‐AD measures, patient‐ and proxy‐reported scores had moderate correlation (*r* = 0.57, *P* < 0.0001). An interaction analysis found that the only variables significantly associated with this correlation were the comorbidities score (*P* = 0.04), number of medications (*P* = 0.04), and anticholinergic burden (*P* = 0.03). Other variables such as age, disease duration, dementia severity, and scores on other patient and caregiver measures had no significant correlation (Table S1 in supporting information).

### 3.2 Patient features associated with patient‐ and proxy‐reported quality of life

Worse (lower) patient‐reported QoL correlated with daytime sleepiness, autonomic symptom burden, and behavioral symptoms (*n* = 61, Table 3). None of the 19 autonomic symptoms assessed by the Autonomic Systems Checklist were significantly correlated with patient‐reported QoL when evaluated individually as dichotomous variables (data not shown). When participants were dichotomized to moderate (*n* = 52) versus severe (*n* = 9) dementia using the QDRS score, worse patient‐reported QoL correlated with LBCRS score (−0.38, *P* = 0.037) in addition to daytime sleepiness, autonomic symptom burden, and behavioral symptoms in the moderate group. No measure significantly correlated with patient‐reported QoL in the severe dementia cohort, but there were only nine participants (Table S2 in supporting information). In a multivariable analysis in which daytime sleepiness, autonomic symptom burden, and behavioral symptoms were included in a single linear regression model as the initial independent variables for a backward model selection, daytime sleepiness and autonomic symptom burden remained in the final model, but not the behavioral symptoms (Table 4).

**TABLE 3:** Univariate correlations with patient‐ and proxy‐reported quality of life.

	Patient‐completed QoL‐AD (*n* = 61)	Proxy‐completed QoL‐AD (*n* = 146)
Patient age	0.24 (*P* = 0.19)	0.006 (*P* = 0.95)
Patient gender	*P* = 0.97	*P* = 0.38
Disease duration at baseline	−0.06 (*P* = 0.79)	0.009 (*P* = 0.95)
LBCRS score	−0.32 (*P* = 0.058)	−0.13 (*P* = 0.30)
QDRS score	−0.26 (*P* = 0.15)	**−0.44 (*P* < 0.0001)**
CDR‐sum of boxes	−0.12 (*P* = 0.60)	**−0.34 (*P* = 0.0001)**
Fluctuation severity	−0.26 (*P* = 0.15)	−0.15 (*P* = 0.23)
RBD symptoms present	*P* = 0.99	*P* = 0.81
Hallucinations present	*P* = 0.90	*P* = 0.61
Excessive daytime sleepiness (ESS score)	**−0.36 (*P* = 0.022)**	**−0.27 (*P* = 0.0039)**
Alertness (Mayo Sleep Questionnaire)	**0.37 (*P* = 0.022)**	**0.36 (*P* = 0.0001)**
Parkinsonism (at least one reported feature)	*P* = 0.79	*P* = 0.77
Parkinsonism (all four reported features of tremor, slowness, rigidity, postural instability)	*P* = 0.69	*P* = 0.75
Autonomic symptoms (total score)	**−0.38 (*P* = 0.022)**	−0.086 (*P* = 0.52)
Orthostatic hypotension present	*P* = 0.30	*P* = 0.64
NPI‐Q severity score	**−0.38 (*P* = 0.022)**	**−0.38 (*P* < 0.0001)**
ADL score	−0.18 (*P* = 0.37)	**−0.24 (*P* = 0.014)**
# of medications	0.12 (*P* = 0.59)	0.053 (*P* = 0.64)
Parkinsonism medications (taking)	*P* = 0.59	*P* = 0.58
Cholinesterase inhibitor (taking)	*P* = 0.61	***P* = 0.024**
Antipsychotic medication (taking)	*P* = 0.86	*P* = 0.37
Antidepressant/anti‐anxiety agent (taking)	*P* = 0.37	*P* = 0.52
Anticholinergic Cognitive Burden scale score	0.041 (*P* = 0.86)	−0.11 (*P* = 0.37)
Charlson Comorbidity Index	0.13 (*P* = 0.59)	−0.057 (*P* = 0.64)

*Notes*: *P*‐values corrected for multiple comparisons using the false discovery rate method. Bold values indicate *P* < 0.05. Correlations and *P* values provided for continuous variables; only *P* values provided for categorical values.

Abbreviations: ADL, activities of daily living; CDR, Clinical Dementia Rating; ESS, Epworth Sleepiness Scale; LBCRS, Lewy Body Composite Risk Score; NPI‐Q, Neuropsychiatric Inventory Questionnaire; QDRS, Quick Dementia Rating System score; QoL‐AD, Quality of Life in Alzheimer's Disease scale; RBD, REM sleep behavior disorder.

**TABLE 4:** Final multivariable quality of life models.

QoL‐AD score type	Variable group	Adjusted R‐squared	Intercept	ASC score	ESS score	MSQ alertness	NPI‐Q total score	QDRS score	Caregiver QoL score
Patient QoL‐AD (*n* = 61)	Patient	0.23	35.26 (*P* < 0.0001)	−0.65 (*P* = 0.0023)		0.86 (*P* = 0.012)			
	Caregiver	N/A							N/A
Proxy QoL‐AD (*n* = 146)	Patient	0.34	33.71 (*P* < 0.0001)			0.65 (*P* = 0.0005)	−0.08 (*P* < 0.0001)	−0.28 (*P* = 0.0014)	
	Caregiver	0.24							0.44 (*P* < 0.0001)
Proxy QoL‐AD with matching patient QoL‐AD (*n* = 61)	Patient	0.37	42.17 (*P* < 0.0001)		−0.27 (*P* = 0.014)		−0.095 (*P* = 0.001)	−0.29 (*P* = 0.047)	
	Caregiver	N/A							N/A
Proxy QoL‐AD without matching patient QoL‐AD (*n* = 85)	Patient	0.26	36.99 (*P* < 0.0001)				−0.076 (*P* = 0.0004)	−0.31 (*P* = 0.0021)	
	Caregiver	0.37							0.53 (*P* < 0.0001)

Abbreviations: ASC, Autonomic Symptom Checklist; ESS, Epworth Sleepiness Scale; MSQ, Mayo Sleep Questionnaire; NPI‐Q, Neuropsychiatric Inventory Questionnaire; QDRS, Quick Dementia Rating System; QoL‐AD, Quality of Life in Alzheimer's Disease scale.

Worse proxy QoL ratings (*n* = 146) correlated with worse dementia severity, daytime sleepiness, behavioral symptoms, an inability to complete ADLs, and whether patient was taking a cholinesterase inhibitor (higher QoL‐AD scores in the group taking a cholinesterase inhibitor; Table 3). When participants were dichotomized to moderate (*n* = 104) versus severe (*n* = 42) dementia using the QDRS score, worse proxy‐reported QoL correlated with worse dementia severity, daytime sleepiness, and behavioral symptoms in the moderate group, but not ADL score. No measure significantly correlated with proxy‐reported QoL in the severe dementia cohort (*n* = 42; Table S2). In the multivariable analysis, dementia severity, daytime sleepiness, and behavioral symptoms remained in the final model, but not ability to complete ADLs or whether patient was taking a cholinesterase inhibitor (Table 4).

Proxy ratings were also assessed separately for the dyads in which the person with DLB and caregiver proxy both completed the QoL‐AD versus the dyads with only a proxy‐completed QoL‐AD. In dyads with both patient and proxy ratings (*n* = 61), worse proxy ratings for patient QoL correlated with dementia severity (by QDRS but not CDR‐SB), daytime sleepiness, and behavioral symptoms, but not ADL dependence. In the cohort with only a proxy‐completed QoL‐AD (*n* = 85), worse QoL‐AD scores correlated with worse dementia severity (by QDRS and CDR‐SB) and behavioral symptoms but not daytime sleepiness or ability to complete ADLs (Table S3 in supporting information). Multivariable analyses were consistent with these results (Table 4).

### 3.3 Caregiver measures and patient quality of life

No measure of the caregiver experience correlated with patient‐completed QoL‐AD scores (Tables 4 and 5). However, better proxy QoL ratings were correlated with higher caregiver QoL, less caregiver depression, lower caregiver burden, lower caregiver grief, higher caregiver self‐efficacy for obtaining respite, and greater reported use of emotion‐focused coping strategies (e.g., acceptance, emotional support, humor, positive reframing, religion; Table 5). Apart from caregiver QoL, however, these correlations were weak, and only caregiver QoL remained in the final multivariable model (Table 4).

**TABLE 5:** Correlations between patient‐ and proxy‐reported quality of life and measures relating to caregiving.

	Patient‐completed QoL‐AD (*n* = 61)	Proxy‐completed QoL‐AD (*n* = 146)
Caregiver age	−0.1 (*P* = 0.79)	0.021 (*P* = 0.88)
Caregiver gender	*P* = 0.80	*P* = 0.46
Years spent caregiving	−0.09 (*P* = 0.79)	0.00056 (*P* > 0.99)
Caregiver quality of life (QoL‐AD)	0.14 (0.79)	**0.48 (*P* < 0.001)**
Caregiver depression (CES‐D)	−0.029 (*P* = 0.94)	**−0.25 (*P* = 0.01)**
Caregiver self‐efficacy for caregiving: controlling upsetting thoughts	0.13 (*P* = 0.79)	0.17 (*P* = 0.076)
Caregiver self‐efficacy for caregiving: obtaining respite	−0.076 (*P* = 0.84)	**0.23 (*P* = 0.014)**
Caregiver self‐efficacy for caregiving: responding to disruptive behaviors	−0.0097 (*P* = 0.94)	0.13 (*P* = 0.18)
Perceived social support	0.056 (*P* = 0.84)	0.18 (*P* = 0.053)
Caregiver burden (sZBI)	−0.23 (*P* = 0.37)	**−0.24 (*P* = 0.011)**
Caregiver grief (MMCGI total)	−0.28 (*P* = 0.37)	**−0.28 (*P* = 0.0044)**
Caregiver resilience (total score)	−0.11 (*P* = 0.79)	0.18 (*P* = 0.053)
Brief COPE: Dysfunctional Coping Strategies	−0.012 (*P* = 0.94)	−0.13 (*P* = 0.18)
Brief COPE: Emotion‐Focused Strategies	0.061 (*P* = 0.84)	**0.24 (*P* = 0.011)**
Brief COPE: Problem‐Focused Strategies	0.23 (*P* = 0.37)	0.018 (*P* = 0.88)

*Notes*: *P* values corrected for multiple comparisons using the FDR method. Bold values indicate *P* < 0.05. Correlations and *P* values provided for continuous variables; only *P* values provided for categorical values.

Abbreviations: CES‐D, Center for Epidemiologic Studies Depression Scale; COPE, Coping Orientation to Problems Experienced; MMCGI, Meuser–Marwit Caregiver Grief Inventory—Short Form; QoL‐AD, Quality of Life in Alzheimer's Disease scale; sZBI, shortened Zarit Burden interview.

On a sensitivity analysis, these findings were largely driven by the 85 dyads with only a proxy‐completed QoL‐AD, also consistent with the multivariable analysis. In dyads with both patient and proxy ratings (*n* = 61), proxy QoL ratings were not significantly correlated with any measure of the caregiver experience. In dyads with only a proxy‐completed QoL‐AD (*n* = 85), higher proxy QoL ratings correlated with higher caregiver QoL, less caregiver depression, higher caregiver self‐efficacy for obtaining respite, higher caregiver resilience, and greater use of emotion‐focused coping strategies (Table S4 in supporting information). In the multivariable analysis, only caregiver QoL remained in the final model (Table 4).

## 4 DISCUSSION

This is the first study assessing features correlated with patient‐reported QoL specifically in moderate–advanced DLB, with additional strengths including the use of patient‐ and caregiver/proxy‐reported QoL and the investigation of the interaction of caregiver measures with patient QoL ratings. The study identified that patient versus proxy ratings of patient QoL were only moderately correlated, with higher average patient‐reported QoL versus proxy scores. QoL for individuals with DLB was negatively impacted by daytime sleepiness and behavioral symptoms regardless of whether the patient or caregiver/proxy was assessing patient QoL. Worse patient‐reported QoL also correlated with overall autonomic symptom burden, though not with any given autonomic symptom. Worse proxy‐reported QoL additionally correlated with worse dementia severity and an inability of the person with DLB to complete ADLs. These results were generally consistent across various sensitivity analyses, though ability to complete ADLs did not significantly correlate in all models. Adjusted *R* ^2^ values in the multivariable analyses ranged from 0.23 to 0.37, implying data variability and limited utility over univariate analyses. Patient‐reported QoL did not correlate with caregiver measures. Proxy‐reported QoL‐AD scores aimed to assess the QoL of the individual with DLB; however, these scores correlated with caregiver QoL, depression, burden, grief, self‐efficacy for obtaining respite, and emotion‐focused coping strategies. Statistically significant correlations were weak to moderate across analyses.

The moderate correlation between patient and caregiver QoL‐AD scores in the current analysis is similar to the correlation observed in one prior study.^1^ In that study enrolling individuals with DLB at various stages (*n* = 34, mean MMSE 17.3, range 0–29), correlation between patient‐ and caregiver‐completed QoL‐AD scores was 0.56.^1^ Current findings are also similar to studies enrolling individuals with AD dementia and their caregivers, in which caregivers commonly rated patients’ QoL as worse than patients did themselves and patient–proxy QoL‐AD correlations were weak or moderate.^40^, ^41^, ^42^ This suggests that patient and proxy assessments of patient QoL may measure somewhat different things and/or have different influences (e.g., caregiver experiences influencing proxy ratings). Clinicians and researchers need to account for this when interpreting QoL reports from individuals with DLB versus caregivers/proxies.

Prior studies assessing symptoms correlating with patient‐reported QoL—which enrolled individuals with DLB earlier in their disease course—found that lower QoL in DLB correlated with more depressive symptoms, higher IADL dependence, and constipation.^4^ ^.^ ^5^ The association of patient‐reported QoL with depression is consistent with the association with behavioral symptoms (as assessed by the NPI‐Q) in the current study. The association of constipation with patient‐rated QoL could also be consistent with the current finding of an association with QoL and autonomic symptom burden, though constipation (assessed as yes/no) was not significantly associated with QoL in the current analysis. The previously identified correlation between IADL dependence and QoL in milder DLB cohorts is of limited relevance for the current cohort, as the focus on moderate–advanced DLB means that participants were already likely dependent in IADLs.

In studies assessing QoL using proxy reports, correlates of QoL for individuals with DLB included dementia severity, neuropsychiatric/behavioral symptoms, dependence in IADLs/ADLs, and whether the person with DLB lived with the primary caregiver.^1^, ^7^ A study combining caregivers for individuals with DLB, AD dementia, and Parkinson's disease dementia identified that proxy‐rated QoL correlated with patient age, disease duration, dementia severity, and caregiver relationship. Most of the individuals in the current study lived with their caregiver (88%) and had spousal caregivers (81%), so living situation and caregiver relationship could not be readily assessed as potential QoL contributors. While age and disease duration were not significantly correlated with QoL in the current analysis, prior proxy‐rated QoL associations with dementia severity, neuropsychiatric/behavioral symptoms, and dependence in IADLs/ADLS are consistent with current findings. Additionally, daytime sleepiness and use of a cholinesterase inhibitor were associated with QoL in the main analyses. The correlation of cholinesterase inhibitor use with proxy‐reported QoL was not replicated in the sensitivity analyses. However, this finding could reflect meta‐analysis results suggesting that cholinesterase inhibitors may help behavioral symptoms in Lewy body dementia.^43^ Daytime sleepiness was associated with QoL across many of the analyses, including for patient‐reported QoL scores.

The finding that worse patient‐reported QoL correlated with daytime sleepiness, autonomic symptom burden, and behavioral symptoms suggests that some currently available pharmacologic strategies could potentially improve the QoL of individuals with DLB. A recent review of Lewy body dementia management outlined numerous strategies for addressing the behavioral, autonomic (e.g., orthostatic hypotension, gastrointestinal dysfunction, urinary symptoms), and sleep concerns that are commonly present in DLB.^44^ No single autonomic feature correlated with patient‐related QoL, however, so the QoL benefit of addressing any one symptom is uncertain. Additionally, options for some symptoms associated with QoL, such as daytime sleepiness, remain limited. Only one single‐arm pilot study investigated treatment of daytime sleepiness in individuals with DLB and hypersomnia.^45^ Armodafinil use in that study resulted in no significant differences in patient QoL, whether self‐ or caregiver reported. Thus, while there may be the opportunity to target symptoms associated with patient QoL in DLB, there is also a clear need for better symptomatic approaches.

This is the first study to assess the correlation between caregiver measures and patient QoL in DLB. No caregiver measures significantly correlated with patient‐reported QoL, whereas caregiver QoL, depression, burden, grief, self‐efficacy for obtaining respite, and emotion‐focused coping strategies correlated with proxy ratings, particularly in the group with only proxy‐rated QoL scores. These are associations, however, and causality is uncertain, including the direction of any possible causality. It is plausible that if individuals with DLB have better QoL, caregiving is easier, and caregivers are less burdened and depressed and have better personal QoL. It is also plausible that the caregivers’ own experiences influence how they interpret the QoL of their loved ones with DLB, with caregivers who are more stressed and burdened and who have worse personal QoL presuming this is also the case for individuals with DLB. It is also possible that less stressed and depressed caregivers improve the QoL of the care recipients and that this association was missed in the patient QoL‐AD cohort due to the smaller sample size or other reasons. This possibility is supported by limited prior research suggesting that caregiver interventions in dementia have the possibility of improving patient QoL.^46^

Strengths of the current analysis include the use of both patient‐ and caregiver/proxy‐reported measures of QoL for individuals with moderate–advanced DLB, consideration of patient ratings in assessing features associated with QoL, and the investigation of the interaction of caregiver measures with patient QoL. Limitations include the use of patient‐, caregiver‐, or clinician‐reported DLB diagnosis supported by a positive LBCRS screen rather than formal application of DLB criteria. This approach was chosen to accommodate the volunteer cohort recruited through advocacy organizations (rather than specialty clinics) while using the same protocol across all participants. Additionally, the design emphasized generalizability to all individuals with a clinical diagnosis of DLB while acknowledging that this can involve misdiagnoses and individuals with relevant comorbidities.

Not all individuals with DLB in the study completed the QoL‐AD (either by choice or due to disease severity) and thus patient‐reported findings may not fully generalize. Specific reasons for non‐completion were not collected. When dementia was dichotomized as moderate versus severe in post hoc analyses, the severe group was relatively small, limiting the ability to detect significant correlations. The QoL‐AD was the only patient‐completed scale, so correlations were made with caregiver‐reported measures of patient symptoms. Due to the fact that most visits were remote, there was no physical measurement of parkinsonism (i.e., the Unified Parkinson's Disease Rating Scale) for the majority of participants. Participants were United States‐based and largely identified as White non‐Hispanic, with more male individuals with DLB and more women caregivers. While this is consistent with the majority of currently available DLB research, implications across sexes and for other cultures and geographies are uncertain.

In conclusion, this study demonstrated that ratings of QoL for individuals with DLB and their caregivers have only moderate correlation, emphasizing the importance of querying both individuals in clinical care and research settings. While it can be easier to make decisions based on caregiver report given the cognitive impairment, bradyphrenia, overall slowness, and soft speech in DLB, caregiver assessments do not fully represent experiences as related by individuals with DLB themselves. It is also important to recognize that patient and proxy assessments of patient QoL may measure different things and/or have different influences (e.g., caregiver experiences influencing proxy ratings) and to account for this when interpreting QoL scores. While some drivers of QoL overlapped between reporters (behavioral symptoms, daytime sleepiness), other features correlating with QoL were distinct (e.g., autonomic symptoms, dementia severity) and this may also reflect differing patient versus proxy experiences. The fact that some features associated with QoL in moderate–advanced DLB have pharmacologic treatments emphasizes the importance of identifying and attempting to treat these symptoms in clinical care. There is also clearly a need, however, for additional studies regarding QoL along the continuum of DLB and for pharmacologic and non‐pharmacologic therapeutic approaches that can improve QoL.

## CONFLICT OF INTEREST STATEMENT

M.J.A. is employed by the University of Florida. She receives research support from the NIH (R01AG068128, P30AG047266, R01NS121099, R44AG062072), the Florida Department of Health (grant 20A08), and as the local PI of a Lewy Body Dementia Association Research Center of Excellence. She serves on the DSMBs for the Alzheimer's Therapeutic Research Institute/Alzheimer's Clinical Trial Consortium and the Alzheimer's Disease Cooperative Study. She has provided educational content for Medscape. H.L.P. receives funding from the NIA (1P30AG053760) and the Carl Rinne Lewy Body Dementia Initiative, and is the local PI of a Lewy Body Dementia Association Research Center of Excellence. C.M. receives research support from ACL/DHHS (90ALGG0014‐01‐00), NIA/NIH (2SB1AG037357‐04A1, R01‐AG‐054435), HRSA (U1QHP287440400), and DoD (AZ190036). She is the local PI of a Lewy Body Dementia Association Research Center of Excellence. J.A.F. receives research support from the NIA (U01NS100620, R01AG068128, R43AG65088). AL receives research support from the NIA (P30AG62677, R43AG65088). She is part of the Lewy Body Dementia Association Coordinating Center Research Center of Excellence and serves on the Scientific Advisory Board of the Lewy Body Dementia Association. B.F.B. has served as an investigator for clinical trials sponsored by Biogen, Alector, and Transposon. He serves on the Scientific Advisory Board of the Lewy Body Dementia Association, Association for Frontotemporal Degeneration, and Tau Consortium. He is the site PI of a Lewy Body Dementia Association Research Center of Excellence program, as well as coordinating center PI of the program. He receives research support from the NIH, the American Brain Foundation, the Mayo Clinic Dorothy and Harry T. Mangurian Jr. Lewy Body Dementia Program, the Little Family Foundation, and the Ted Turner and Family Functional Genomics Program. J.E.G. is the creator of the QDRS and the LBCRS. He receives research support from the National Institutes of Health (R01NS101483, R01NS101483S1, R01AG071514, R01AG071514S1, R56AG074889, P01AG066584, R01AG071643, R01AG069765, R01AG057681, P30AG059295, RF1AG075901, R01AG078214, S06GM142115, R01AG056531, R01AG068128, P30AG066506) and as the local PI of a Lewy Body Dementia Association Research Center of Excellence. He serves as a consultant for Alpha Cognition, Biogen, Eisai, Eli Lilly, GE Healthcare, Genentech, and Roche. He is an investigator on clinical trials with CND Life Sciences, Cognition Therapeutics, and EIP Pharma. He serves as Chief Scientific Officer for Cognivue. He serves on the Board of Directors for the Lewy Body Dementia Association, Lewy Body Dementia Resource Center, and South Florida Chapter of the Alzheimer's Association (all unpaid). A.S.T. is an employee of the Lewy Body Dementia Association. B.L., K.S., S.M.M., L.F., and Z.L. have no conflicts of interest to report. Author disclosures are available in the supporting information.

## Supporting information

Supporting Information

Supporting Information

## References

[1] Boström F , Jönsson L , Minthon L , Londos E . Patients with dementia with Lewy bodies have more impaired quality of life than patients with Alzheimer disease. Alzheimer Dis Assoc Disord. 2007;21:150‐154. 17545741 10.1097/WAD.0b013e318065c4a9

[2] Thomas P , Lalloue F , Preux PM , et al. Dementia patients caregivers quality of life: the PIXEL study. Int J Geriatr Psychiatry. 2006;21:50‐56. 16323256 10.1002/gps.1422

[3] Figari‐Jordan R , Anderson K , Gruber‐Baldini A , et al. Comparison of quality of life and disability in three different dementias (P07.180). Neurology. 2012;78:P07180.

[4] van de Beek M , van Steenoven I , Ramakers I , et al. Trajectories and determinants of quality of life in dementia with Lewy bodies and Alzheimer's disease. J Alzheimers Dis. 2019;70:389‐397. 31177218 10.3233/JAD-190041 PMC6839497

[5] van de Beek M , van Steenoven I , van der Zande JJ , et al. Characterization of symptoms and determinants of disease burden in dementia with Lewy bodies: dEvELOP design and baseline results. Alzheimers Res Ther. 2021;13:53. 33637117 10.1186/s13195-021-00792-w PMC7908769

[6] van de Beek M , van Unnik A , van Steenoven I , et al. Disease progression in dementia with Lewy bodies: a longitudinal study on clinical symptoms, quality of life and functional impairment. Int J Geriatr Psychiatry. 2022;37(12). doi:10.1002/gps.5839 PMC9828829 36403133

[7] Armstrong M , Monari E , Almeida L , McFarland N , Malaty I , Okun M . Initial patterns and correlates of quality of life in dementia with Lewy bodies (P6.234). Neurology. 2016;86:p6234.

[8] Armstrong MJ , Gamez N , Alliance S , et al. Research priorities of caregivers and individuals with dementia with Lewy bodies: an interview study. PLoS ONE. 2020;15:e0239279. 33027276 10.1371/journal.pone.0239279 PMC7540843

[9] Armstrong MJ , Paulson HL , Maixner SM , et al. Protocol for an observational cohort study identifying factors predicting accurately end of life in dementia with Lewy bodies and promoting quality end‐of‐life experiences: the PACE‐DLB study. BMJ Open. 2021;11:e047554. 10.1136/bmjopen-2020-047554 PMC8160156 34039578

[10] Galvin JE . The Quick Dementia Rating System (QDRS): a rapid dementia staging tool. Alzheimers Dement (Amst). 2015;1:249‐259. 26140284 10.1016/j.dadm.2015.03.003 PMC4484882

[11] Lacruz M , Emeny R , Bickel H , Linkohr B , Ladwig K . Feasibility, internal consistency and covariates of TICS‐m (telephone interview for cognitive status‐modified) in a population‐based sample: findings from the KORA‐Age study. Int J Geriatr Psychiatry. 2013;28:971‐978. 23242951 10.1002/gps.3916

[12] Galvin JE . Improving the clinical detection of Lewy body dementia with the Lewy Body Composite Risk Score. Alzheimers Dement (Amst). 2015;1:316‐324. 26405688 10.1016/j.dadm.2015.05.004 PMC4576496

[13] Thorgrimsen L , Selwood A , Spector A , et al. Whose quality of life is it anyway? The validity and reliability of the Quality of Life‐Alzheimer's Disease (QoL‐AD) scale. Alzheimer Dis Assoc Disord. 2003;17:201‐208. 14657783 10.1097/00002093-200310000-00002

[14] Cummings JL , Mega M , Gray K , Rosenberg‐Thompson S , Carusi DA , Gornbein J . The Neuropsychiatric Inventory: comprehensive assessment of psychopathology in dementia. Neurology. 1994;44:2308‐2314. 7991117 10.1212/wnl.44.12.2308

[15] Kaufer DI , Cummings JL , Ketchel P , et al. Validation of the NPI‐Q, a brief clinical form of the Neuropsychiatric Inventory. J Neuropsychiatry Clin Neurosci. 2000;12:233‐239. 11001602 10.1176/jnp.12.2.233

[16] Ferman TJ , Smith GE , Boeve BF , et al. DLB fluctuations: specific features that reliably differentiate DLB from AD and normal aging. Neurology. 2004;62:181‐187. 14745051 10.1212/wnl.62.2.181

[17] Walker MP , Ayre GA , Cummings JL , et al. The Clinician Assessment of Fluctuation and the One Day Fluctuation Assessment Scale. Two methods to assess fluctuating confusion in dementia. Br J Psychiatry. 2000;177:252‐256. 11040887 10.1192/bjp.177.3.252

[18] Boeve BF , Molano JR , Ferman TJ , et al. Validation of the Mayo Sleep Questionnaire to screen for REM sleep behavior disorder in an aging and dementia cohort. Sleep Med. 2011;12:445‐453. 21349763 10.1016/j.sleep.2010.12.009 PMC3083495

[19] Johns MW MW . A new method for measuring daytime sleepiness: the Epworth sleepiness scale. Sleep. 1991;14:540‐545. 1798888 10.1093/sleep/14.6.540

[20] Galvin JE , Chrisphonte S , Cohen I , et al. Characterization of dementia with Lewy bodies (DLB) and mild cognitive impairment using the Lewy body dementia module (LBD‐MOD). Alzheimers Dement. 2021;17:1675‐1686. 33793069 10.1002/alz.12334 PMC8484363

[21] Morris JN , Fries BE , Morris SA . Scaling ADLs within the MDS. J Gerontol A Biol Sci Med Sci. 1999;54:M546‐553. 10619316 10.1093/gerona/54.11.m546

[22] Mitchell SL , Miller SC , Teno JM , Davis RB , Shaffer ML . The advanced dementia prognostic tool (ADEPT): a risk score to estimate survival in nursing home residents with advanced dementia. J Pain Symptom Manage. 2010;40:639‐651. 20621437 10.1016/j.jpainsymman.2010.02.014 PMC2981683

[23] Charlson ME , Pompei P , Ales KL , MacKenzie CR . A new method of classifying prognostic comorbidity in longitudinal studies: development and validation. J Chronic Dis. 1987;40:373‐383. 3558716 10.1016/0021-9681(87)90171-8

[24] Williams A , Sera L , McPherson ML . Anticholinergic burden in hospice patients with dementia. Am J Hosp Palliat Care. 2019;36:222‐227. 30213190 10.1177/1049909118800281

[25] Kolanowski A , Fick DM , Campbell J , Litaker M , Boustani M . A preliminary study of anticholinergic burden and relationship to a quality of life indicator, engagement in activities, in nursing home residents with dementia. J Am Med Dir Assoc. 2009;10:252‐257. 19426941 10.1016/j.jamda.2008.11.005 PMC2735136

[26] Lewinsohn PM , Seeley JR , Roberts RE , Allen NB . Center for Epidemiologic Studies Depression Scale (CES‐D) as a screening instrument for depression among community‐residing older adults. Psychol Aging. 1997;12:277‐287. 9189988 10.1037//0882-7974.12.2.277

[27] Bédard M , Molloy DW , Squire L , Dubois S , Lever JA , O'Donnell M . The Zarit Burden Interview: a new short version and screening version. Gerontologist. 2001;41:652‐657. 11574710 10.1093/geront/41.5.652

[28] Marwit SJ , Meuser TM . Development of a short form inventory to assess grief in caregivers of dementia patients. Death Stud. 2005;29:191‐205. 15816111 10.1080/07481180590916335

[29] Zimet GD , Powell SS , Farley GK , Werkman S , Berkoff KA . Psychometric characteristics of the Multidimensional Scale of Perceived Social Support. J Pers Assess. 1990;55:610‐617. 2280326 10.1080/00223891.1990.9674095

[30] Hardan‐Khalil K , Mayo AM . Psychometric properties of the multidimensional scale of perceived social support. Clin Nurse Spec. 2015;29:258‐261. 26258831 10.1097/NUR.0000000000000148

[31] Wagnild GM , Young HM . Development and psychometric evaluation of the Resilience Scale. J Nurs Meas. 1993;1:165‐178. 7850498

[32] Steffen AM , McKibbin C , Zeiss AM , Gallagher‐Thompson D , Bandura A . The revised scale for caregiving self‐efficacy: reliability and validity studies. The J Gerontol B. 2002;57:P74‐P86. 10.1093/geronb/57.1.p74 11773226

[33] Steffen AM , Gallagher‐Thompson D , Arenella KM , et al. Validating the revised scale for caregiving self‐efficacy: a cross‐national review. Gerontologist. 2019;56:e325‐e342. 10.1093/geront/gny004 29546334

[34] Charles SC . You want to measure coping but your protocol's too long: consider the Brief COPE. Int J Behavn Med. 1997;4:92‐100. 10.1207/s15327558ijbm0401_6 16250744

[35] Cooper C , Katona C , Livingston G . Validity and reliability of the brief COPE in carers of people with dementia: the LASER‐AD Study. J Nerv Ment Dis. 2008;196:838‐843. 19008735 10.1097/NMD.0b013e31818b504c

[36] Harris PA , Taylor R , Thielke R , Payne J , Gonzalez N , Conde JG . Research electronic data capture (REDCap) – a metadata‐driven methodology and workflow process for providing translational research informatics support. J Biomed Inform. 2009;42:377‐381. 18929686 10.1016/j.jbi.2008.08.010 PMC2700030

[37] Harris PA , Taylor R , Minor BL , et al. The REDCap consortium: building an international community of software platform partners. J Biomed Inform. 2019;95:103208. 31078660 10.1016/j.jbi.2019.103208 PMC7254481

[38] McHugh ML . Interrater reliability: the kappa statistic. Biochem Med (Zagreb). 2012;22:276‐282. 23092060 PMC3900052

[39] Benjamini Y , Hochberg Y . Controlling the false discovery rate: a practical and powerful approach to multiple testing. J R Stat Soc B (Methodological). 1995;57:289‐300.

[40] Kahle‐Wrobleski K , Ye W , Henley D , et al. Assessing quality of life in Alzheimer's disease: implications for clinical trials. Alzheimers Dement (Amst). 2017;6:82‐90. 28229126 10.1016/j.dadm.2016.11.004 PMC5312555

[41] Zucchella C , Bartolo M , Bernini S , Picascia M , Sinforiani E . Quality of life in Alzheimer disease: a comparison of patients' and caregivers' points of view. Alzheimer Dis Assoc Disord. 2015;29:50‐54. 24936799 10.1097/WAD.0000000000000050

[42] Karttunen K , Karppi P , Hiltunen A , et al. Neuropsychiatric symptoms and quality of life in patients with very mild and mild Alzheimer's disease. Int J Geriatr Psychiatry. 2011;26:473‐482. 21445998 10.1002/gps.2550

[43] Meng YH , Wang PP , Song YX , Wang JH . Cholinesterase inhibitors and memantine for Parkinson's disease dementia and Lewy body dementia: a meta‐analysis. Exp Ther Med. 2019;17:1611‐1624. 30783428 10.3892/etm.2018.7129 PMC6364145

[44] Taylor JP , McKeith IG , Burn DJ , et al. New evidence on the management of Lewy body dementia. Lancet Neurol. 2020;19:157‐169. 31519472 10.1016/S1474-4422(19)30153-X PMC7017451

[45] Lapid MI , Kuntz KM , Mason SS , et al. Efficacy, safety, and tolerability of armodafinil therapy for hypersomnia associated with dementia with Lewy bodies: a pilot study. Dement Geriatr Cogn Disord. 2017;43:269‐280. 28448998 10.1159/000471507 PMC5503747

[46] Cooper C , Mukadam N , Katona C , et al. Systematic review of the effectiveness of non‐pharmacological interventions to improve quality of life of people with dementia. Int Psychogeriatr. 2012;24:856‐870. 22244371 10.1017/S1041610211002614

