# Supplementary material for: Patient‐ and proxy‐reported quality of life in advanced dementia with Lewy bodies
Source: Alzheimers Dement. 2024 Feb 23;20(4):2719–30. doi: 10.1002/alz.13745 (PMC11032544; doi:10.1002/alz.13745)
Supplement: Supplementary file 1 — Supporting Information [file ALZ-20-2719-s002.docx]

APPENDIX

Appendix Table A.1 Interaction Analysis for Correlation between Patient- and Proxy-Rated QoL-AD (Unadjusted p)

| Variable | p-Value | +/- |
| --- | --- | --- |
| Patient Age (at study visit) | 0.2324 | N/A |
| Disease Duration (Years) | 0.5917 | N/A |
| Caregiver Age (at study visi | 0.2190 | N/A |
| LBCRS Score | 0.8801 | N/A |
| Fluctuations Severity Score | 0.1802 | N/A |
| Total QDRS Score | 0.5209 | N/A |
| CDR-SB | 0.8318 | N/A |
| TICS-m Score | 0.7567 | N/A |
| MSQ Alertness | 0.8546 | N/A |
| NPI Severity Score | 0.5423 | N/A |
| NPI Total Score | 0.4745 | N/A |
| ESS Score | 0.3565 | N/A |
| ASC Score | 0.2962 | N/A |
| CES-D Score | 0.9199 | N/A |
| ADL Score | 0.0895 | N/A |
| Number of Medications | **0.0350** | **-** |
| Anticholinergic Burden Scale | **0.0327** | **-** |
| Charlson Comorbidity Index | **0.0419** | **-** |
| QoL-AD (Proxy) Total Score | 0.6054 | N/A |
| Self-Efficacy: Obtaining Respite | 0.1354 | N/A |
| Self-Efficacy: Responding to Disruptive Behaviors | 0.2319 | N/A |
| Self-Efficacy: Controlling Upsetting Thoughts | 0.3671 | N/A |
| Perceived Social Support Score | 0.1882 | N/A |
| sZBI Score | 0.6607 | N/A |
| MMCGI: Total Grief Level | 0.2970 | N/A |
| Resilience Total Score | 0.6319 | N/A |
| Brief COPE: Emotion-Focused | 0.0923 | N/A |
| Brief COPE: Problem-Focused | 0.5946 | N/A |
| Brief COPE: Dysfunctional Coping | 0.4735 | N/A |
| Patient Gender | 0.0764 | N/A |
| Caregiver Gender | 0.6750 | N/A |
| MSQ RBD | 0.7801 | N/A |
| NPI-Q Hallucinations Present | 0.1357 | N/A |
| Parkinsonism (LBCRS - All) | 0.0571 | N/A |
| Orthostatic Hypotension | 0.7617 | N/A |
| Use of Parkinson Medication | 0.8075 | N/A |
| Use of Antidepressant Medication | 0.5129 | N/A |
| Use of Antipsychotic Medication | 0.3670 | N/A |
| Acetylcholinesterase Inhibition Use | 0.2351 | N/A |
| Excessive Sleepiness by ESS | 0.6395 | N/A |
| Fluctuations Present | 0.8545 | N/A |
| Dementia Severity | 0.4968 | N/A |

N/A: Not applicable

QoL-AD: Quality of Life – Alzheimer Disease scale; LBCRS: Lewy Body Composite Risk Score; QDRS: Quick Dementia Rating System score; CDR-SB: Clinical Dementia Rating Sum of Boxes; TICS-m: Telephone Interview for Cognitive Status – modified; MSQ: Mayo Sleep Questionnaire; NPI-Q: Neuropsychiatric Inventory Questionnaire; ESS: Epworth Sleepiness Scale; ASC: Autonomic Symptoms Checklist; CES-D: Center for Epidemiologic Studies Depression Scale; ADL: Activities of daily living; sZBI: shortened Zarit Revised Burden interview; MMCGI: Meuser-Marwit Caregiver Grief Inventory - Short Form; RBD: REM Sleep Behavior Disorder

Table A.2. Univariate Correlations with Patient-Reported Quality of Life in Moderate versus Advanced Dementia

|  | Patient-Reported QoL (n=61) | | Proxy-Reported QoL for All Participants (n=146) | |
| --- | --- | --- | --- | --- |
|  | Moderate dementia (n=52) | Severe dementia (n=9) | Moderate Dementia (n=104) | Severe Dementia (n=42) |
| Patient age | 0.31, p=0.10 | 0.13, p=1.00 | 0.08, p=0.76 | -0.08, p=0.8 |
| Patient gender | p=0.92 | p=1.00 | p=0.97 | p=0.37 |
| Disease duration at baseline | -0.10, p=0.77 | -0.11, p=1.00 | 0.05, p=0.89 | 0.05, p=0.78 |
| LBCRS score | **-0.38, p=0.037** | 0.22, p=1.00 | -0.04, p=0.89 | -0.27, p=0.37 |
| QDRS score | -0.21, p=0.32 | 0.38, p=1.00 | **-0.29, p=0.032** | -0.35, p=0.23 |
| CDR-sum of boxes | -0.02, p=0.92 | 0.16, p=1.00 | -0.14, p=0.41 | -0.14, p=0.78 |
| Fluctuation severity | -0.26, p=0.18 | -0.41, p=1.00 | -0.19, p=0.18 | 0.039, p=0.91 |
| RBD symptoms present | p=0.91 | p=1.00 | p=0.97 | p=0.91 |
| Hallucinations present | p=0.86 | p=1.00 | p=0.81 | p=0.78 |
| Excessive daytime sleepiness (ESS score) | **-0.37, p=0.039** | -0.21, p=1.00 | -0.21, p=0.13 | 0.18, p=0.59 |
| Alertness (Mayo Sleep Questionnaire) | 0.35, p=0.06 | 0.30, p=1.00 | **0.29, p=0.034** | 0.38, p=0.23 |
| Parkinsonism (all four reported features of tremor, slowness, rigidity, postural instability) | p=0.77 | p=1.00 | p=0.97 | p=0.89 |
| Autonomic symptoms (total score) | **-0.47, p=0.007** | -0.03, p=1.00 | 0.01, p=0.97 | 0.01, p=0.37 |
| Orthostatic hypotension present | p=0.18 | p=1.00 | p=0.97 | p=0.57 |
| NPI-Q severity score | **-0.41, p=0.027** | -0.19, p=1.00 | **-0.41, p=0.0002** | -0.45, p=0.32 |
| ADL score | -0.24, p=0.21 | 0.63, p=0.40 | 0.003, p=0.98 | -0.11, p=0.78 |
| # of medications | 0.06, p=0.86 | 0.26, p=1.00 | 0.04, p=0.89 | 0.004, p=0.98 |
| Parkinsonism medications (taking) | p=0.33 | p=1.00 | p=0.55 | p=0.37 |
| Cholinesterase inhibitor (taking) | p=0.77 | p=1.00 | p=0.075 | p=0.91 |
| Antipsychotic medication (taking) | p=0.86 | p=0.40 | p=0.86 | p=0.78 |
| Antidepressant/anti-anxiety agent (taking) | p=0.65 | p=0.85 | p=0.76 | p=0.88 |
| Anticholinergic Cognitive Burden scale score | -0.02, p=0.92 | 0.65, p=0.40 | -0.02, p=0.97 | -0.13, p=0.78 |
| Charlson Comorbidity Index | 0.01, p=0.94 | 0.73, p=0.40 | -0.05, p=0.89 | 0.02, p=0.94 |

P-values corrected for multiple comparisons using the FDR method. Bold values indicate p<0.05. Correlations and p-values provided for continuous variables; only p-values provided for categorical values.

QoL-AD: Quality of Life – Alzheimer Disease scale; LBCRS: Lewy Body Composite Risk Score; QDRS: Quick Dementia Rating System score; CDR: Clinical Dementia Rating; RBD: REM sleep behavior disorder; NPI-Q: Neuropsychiatric Inventory Questionnaire; ADL: Activities of daily living; ESS: Epworth Sleepiness Scale

Table A.3. Univariate Correlations with Proxy-Related Quality of Life in Dyads with Both Patient- and Proxy-Reported Quality of Life Versus Dyads with Only Proxy-Reported Quality of Life

|  | Proxy-Completed QoL-AD in Dyads where Patients also Completed the QoL-AD (n=61) | Proxy-completed QoL-AD in Dyads where Only the Caregiver Completed the QoL-AD (n=85) |
| --- | --- | --- |
| Patient age | 0.087 (p=0.76) | 0.039 (p=0.82) |
| Patient gender | p=0.75 | p=0.31 |
| Disease duration at baseline | 0.072 (p=0.84) | -0.0043 (p=0.98) |
| LBCRS score | -0.22 (p=0.29) | -0.092 (p=0.54) |
| QDRS score | **-0.42 (p=0.0082)** | **-0.39 (p=0.0033)** |
| CDR-sum of boxes | -0.27 (p=0.15) | **-0.31 (p=0.019)** |
| Fluctuation severity | -0.17 (p=0.45) | -0.11 (p=0.48) |
| RBD symptoms present | p=0.94 | p=0.98 |
| Hallucinations present | p=0.88 | p=0.35 |
| Excessive daytime sleepiness (ESS score) | **-0.46 (p=0.0082)** | -0.11 (p=0.48) |
| Alertness (Mayo Sleep Questionnaire) | **0.43 (p=0.0082)** | 0.26 (p=0.087) |
| Parkinsonism (at least one reported feature) | p=0.32 | p=0.82 |
| Parkinsonism (all four reported features of tremor, slowness, rigidity, postural instability) | p=0.75 | p=0.74 |
| Autonomic symptoms (total score) | -0.22 (p=0.28) | -0.06 (p=0.71) |
| Orthostatic hypotension present | p=0.94 | p=0.41 |
| NPI-Q severity score | **-0.42 (p=0.0082)** | **-0.35 (p=0.0098)** |
| ADL score | -0.31 (p=0.076) | -0.14 (p=0.41) |
| # of medications | 0.26 (p=0.15) | -0.11 (p=0.48) |
| Parkinsonism medications (taking) | p=0.94 | p=0.48 |
| Cholinesterase inhibitor (taking) | p=0.076 | p=0.3 |
| Antipsychotic medication (taking) | p=0.76 | p=0.11 |
| Antidepressant (taking) | p=0.76 | p=0.69 |
| Anticholinergic Cognitive Burden scale score | 0.18 (p=0.38( | **-0.29 (0.028)** |
| Charlson Comorbidity Index | 0.10 (p=0.75) | -0.11 (p=0.49) |

P-values corrected for multiple comparisons using the FDR method. Bold values indicate p<0.05. Correlations and p-values provided for continuous variables; only p-values provided for categorical values.

LBCRS: Lewy Body Composite Risk Score; QDRS: Quick Dementia Rating System score; CDR: Clinical Dementia Rating; RBD: REM sleep behavior disorder; NPI-Q: Neuropsychiatric Inventory Questionnaire; ADL: Activities of daily living; ESS: Epworth Sleepiness Scale

Table A.4. Univariate Correlations Between Caregiver Measures and Proxy-Related Quality of Life in Dyads with Both Patient- and Proxy-Reported Quality of Life Versus Dyads with Only Proxy-Reported Quality of Life

|  | Proxy-Completed QoL-AD in Dyads where Patients also Completed the QoL-AD (n=61) | Proxy-completed QoL-AD in Dyads where Only the Caregiver Completed the QoL-AD (n=85) |
| --- | --- | --- |
| Caregiver age | -0.021 (p=0.93) | 0.06 (p=0.71) |
| Caregiver gender | p=0.88 | p=0.71 |
| Years spent caregiving | 0.06 (p=0.88) | 0.04 (p=0.74) |
| Caregiver quality of life (QoL-AD) | 0.2 (p=0.62) | **0.61 (p<0.0001)** |
| Caregiver depression (CES-D) | -0.15 (p=0.75) | **-0.31 (p=0.02)** |
| Caregiver self-efficacy for caregiving: controlling upsetting thoughts | 0.09 (p=0.88) | 0.22 (p=0.11) |
| Caregiver self-efficacy for caregiving: obtaining respite | 0.06 (p=0.88) | **0.33 (p=0.02)** |
| Caregiver self-efficacy for caregiving: responding to disruptive behaviors | 0.01 (p=0.93) | 0.22 (p=0.13) |
| Perceived social support | 0.06 (p=0.88) | 0.24 (p=0.08) |
| Caregiver burden (sZBI) | -0.27 (p=0.28) | -0.25 (p=0.08) |
| Caregiver grief (MMCGI total) | -0.31 (p=0.22) | -0.26 (p=0.07) |
| Caregiver resilience (total score) | -0.03 (p=0.93) | **0.35 (p=0.01)** |
| Brief COPE: Dysfunctional Coping Strategies | -0.16 (p=0.75) | -0.12 (p=0.48) |
| Brief COPE: Emotion-Focused Strategies | 0.06 (p=0.88) | **0.34 (p=0.01)** |
| Brief COPE: Problem-Focused Strategies | 0.1 (p=0.88) | 0.01 (p=0.96) |

P-values corrected for multiple comparisons using the FDR method. Bold values indicate p<0.05. Correlations and p-values provided for continuous variables; only p-values provided for categorical values.

QoL-AD: Quality of Life – Alzheimer Disease scale; CES-D: Center for Epidemiologic Studies Depression Scale; sZBI: shortened Zarit Revised Burden interview; MMCGI: Meuser-Marwit Caregiver Grief Inventory - Short Form
